# Processing and Characterization of Air-Sprayed Bismuth Titanate Ultrasonic Transducers

**DOI:** 10.3390/s26061747

**Published:** 2026-03-10

**Authors:** Maryam Ghodousi, Bernhard Tittmann, Cliff J. Lissenden

**Affiliations:** Department of Engineering Science and Mechanics, The Pennsylvania State University, University Park, PA 16802, USA; mpg6051@psu.edu (M.G.); brt4@psu.edu (B.T.)

**Keywords:** ultrasonic transducer, bismuth titanate, air-sprayed transducer, piezoelectric transducer characterization, thermal aging

## Abstract

Transducers for ultrasonic nondestructive evaluation of materials in harsh environments are needed to manage safe operations in a number of industrial applications including power generation, propulsion, and material and chemical processing. Bismuth titanate has a reasonably high Curie temperature and transduces electrical energy into elastic waves and vice versa. Herein, a slurry containing bismuth titanate powder is air-sprayed onto stainless steel substrates, functionalized, and characterized in terms of coating thickness, center frequency and bandwidth, and signal-to-noise ratio. Coatings 40– 70 μm thick had a center frequency of approximately 7 MHz and a broad frequency response range of 3–20 MHz. Transducers were thermally aged at 375 °C for seven days to assess their temperature tolerance. Post-aging analysis revealed a resonance frequency increase, thickness reduction, and microstructural changes, accompanied by a decrease in signal amplitude. Despite these changes, the aged transducers remained operational with good signal-to-noise ratio. Thermal cycling experiments showed that the response of pristine transducers is changed by cycling to 250 °C, while thermally aged transducers exhibited stable ultrasonic performance. Additional experiments on transducers pre-conditioned at 400 °C demonstrated improved thermal resilience after thermal aging at 350 °C. These field deployable air-sprayed BIT transducers are promising candidates for high-temperature NDE applications.

## 1. Introduction

Ultrasonic transducers are used for nondestructive evaluation (NDE) and structural health monitoring (SHM) in industrial environments. Lead zirconate titanate (PZT) ceramics have long served as the active material in ultrasonic transducers for NDE and SHM, owing to their high electromechanical coupling (e.g., $k_{33}$) and large piezoelectric coefficients (e.g., $d_{33}$). Commercial PZT elements exhibit $d_{33}$ values in the range of approximately 330–575 pC/N and $k_{33}$ around 0.7 [1,2]. Despite their excellent piezoelectric properties, conventional PZT ceramics are limited by their Curie temperature (∼350 °C) [3], which limits their operating temperature to around ∼150–200 °C [4]. These limitations, combined with increasing environmental regulations surrounding lead-based materials, underscore the need for alternative piezoelectric materials.

To address these challenges, several alternatives have been investigated, offering higher Curie temperatures, improved thermal stability, and in some cases, environmental benefits. Notable candidates include bismuth titanate (Bi_4_Ti_3_O_12_ or BIT for short), lithium niobate (LiNbO_3_), gallium orthophosphate (GaPO_4_), and aluminum nitride (AlN). Among these, BIT offers a favorable combination of high Curie temperature (650–770 °C) and moderate piezoelectric response ($d_{33}\approx 26$ pC/N) [5]. Early studies, such as [6,7], validated BIT-based transducers for use in harsh environments. BIT is one of the most promising materials for many high-temperature applications [4].

In addition to depoling, conventional ultrasonic transducers face practical challenges at elevated temperatures due to the thermal instability of couplants and bonding agents. Traditionally, a gel or fluid couplant is used between the transducer and the substrate to facilitate efficient transmission of ultrasonic waves. However, at high temperatures, these couplants can lose their effectiveness. The failure of these couplants compromises the functionality of the transducer, particularly in environments where continuous or long-term monitoring is required.

A robust approach to overcome these challenges is to directly deposit transducers on the structure, thereby eliminating the need for couplant and enabling the transducer to stay in place, which is especially valuable in inaccessible or hazardous environments. Air-spray deposition techniques have developed for this purpose. A fundamental advance was the sol-gel spray-on technique pioneered by Barrow et al., who used PZT composites to achieve strong adhesion and crack-free coatings [8,9,10,11]. This process involves combining PZT powder with an organic binder and depositing it on the substrate, followed by a sintering process at above 450 °C to remove organics and achieve durable and flexible piezoelectric films. Later, Kobayashi et al. expanded this approach by incorporating high-temperature piezoelectrics such as LiNbO_3_, BIT, and CaBi_4_Ti_4_O_15_. This innovation enabled transducers capable of operating at temperatures up to 550 °C [12,13,14,15].

Tittmann’s lab at Penn State built on these advances through a series of graduate theses. Searfass evaluated BIT/PZT sol-gel transducers for high-temperature wave generation [16,17] and introduced a mobile sintering method using a blow torch [18]. Later, Orr investigated the effect of bond stiffness on ultrasonic wave transmission at the transducer-substrate interface [19]. Sinding’s research compared a commercial 5 MHz transducer with a BIT spray-on transducer [20]. However, a key limitation of sol-gel-based fabrication is its incompatibility with reactive metal substrates, which are prone to corrosion during the process. Additionally, the requirement for a high-temperature sintering step makes it less suitable for field-deployable applications. To address this challenge, researchers began exploring alternative methods that avoid surface degradation and enable broader applicability. Ledford achieved a significant breakthrough for air-spray deposition by mixing BIT powder with a high-temperature inorganic binder [21]. This approach avoids the limitations of sol-gel methods and provides a durable solution for high-temperature applications. While Ledford introduced the method, Xu’s research focused on optimizing BIT transducer processing [22]. Trivedi further advanced this work by determining the optimal mixture to enhance the consistency and reliability of BIT transducers [23,24]. Table 1 provides a summary of ultrasonic transducer characteristics with a focus on elevated temperature environments.

While significant progress has been made in the development of air-sprayed transducers, their performance after thermal exposure and cycling needs to be established. In addition, field-deployable deposition techniques have not been adequately assessed in terms of the uniformity of the transducer characteristics. The objective of this article is to demonstrate the capabilities of air-sprayed BIT ultrasonic transducers in pulse-echo mode. To do this, we functionalized a reasonably large population (N = 20) of transducers using field deployable methods and evaluated their properties as well as their stability with respect to thermal aging and cycling. Both the BIT material and transducer deposition/functionalization methods are amenable to field installation and service at elevated temperatures. Our findings are important in relation to the use of these transducers for structural health monitoring applications.

The remainder of this article unfolds by detailing the processing methodology used to create the air-sprayed BIT transducers. This is followed by a description of the characterization procedures used to evaluate their properties. The core results are then presented, including the ultrasonic performance of pristine transducers, followed by a series of investigations of the effect of thermal aging, thermal cycling, and pre-conditioning. These sections highlight how thermal exposure influences signal stability and piezoelectric properties. Finally, we conclude with a summary of key findings and implications for high-temperature, field-deployable NDE and SHM applications.

## 2. Materials and Methods

Twenty samples are processed as described in this section, each being a 12.7 mm diameter stainless steel disk with a functionalized BIT transducer 9.5 mm in diameter centered on the disk. Subsequently, the physical characteristics of the transducers are reported.

### 2.1. Processing Transducers

#### 2.1.1. Materials and Slurry Preparation

The backbone of the transducers is 99.9% pure Bi_4_Ti_3_O_12_ powder from Lorad Chemical Corporation (Tampa, FL, USA). Our particle size distribution analysis, conducted using a laser diffraction particle size analyzer (Mastersizer 3000, Malvern Panalytical Ltd., Worcestershire, UK), revealed an average particle size of 4.74 µm and a range of 1.73–37.8 µm. The BIT powder, ceramic binder (Ceramabind 830, Aremco Products Inc., Valley Cottage, NY, USA) and distilled water are mixed in the specific ratio of 1 g of powder, 0.2 mL of binder, and 0.8 mL of water to form a homogeneous slurry, as per Trivedi [24]. The slurry is transferred to the hopper of a portable airbrush sprayer (Master Airbrush G222, Master Airbrush, San Diego, CA, USA) for deposition.

#### 2.1.2. Slurry Deposition

A stainless steel disk (grade 304, mill finish, 12.7 mm diameter and 6.35 mm thick) serves as both the substrate and the bottom electrode for the transducer. The disk surface was prepared by abrading with 2000-grit paper to remove surface imperfections, followed by cleaning with ethanol and acetone to eliminate residual contaminants. The disk is masked to the desired areal extent of the coating. The slurry is air-sprayed with a standard nozzle and 40 kPa pressure onto the substrate in multiple passes. Each layer is approximately 1–3 µm thick and is given time to dry before the next layer is applied. This multi-layer approach minimizes cracking and delamination. Depositing one transducer requires a large number of passes; e.g., 30 layers for a 60 µm thick coating. Comparing the cumulative thickness to the mask thickness helps the operator decide when to stop deposition.

#### 2.1.3. Electrode Deposition

A conductive silver paint (842AR Super Shield, MG Chemicals, Burlington, ON, Canada) is brush-applied to the BIT coating. To achieve consistent electrode geometry and sharp edges, scotch tape masks are prepared using a CO_2_ laser to define a circular region with a diameter of 9.5 mm. This ensures uniform active areas for all transducers in the population, enabling comparisons amongst the transducers. The BIT coating has porosity, thus it is imperative that the silver paste does not wick through the BIT and create a short circuit. Some researchers have used a gold seed layer to seal the BIT surface prior to depositing the silver paste [21], but we did not.

#### 2.1.4. Polarization

Poling is the final step to functionalize the transducer. The piezoelectric properties of the BIT are activated by aligning the internal dipoles with an applied electric field. The sample is enclosed in a cylindrical housing to maintain a uniform temperature during the process. Lead wires are connected to the top electrode and the substrate by manual contact. A traditional oil or vacuum bath was not used because it may be impractical for some field-deposition applications.

Poling was performed using a Trek Model 30/20 high-voltage amplifier system (Trek, Inc., Lockport, NY, USA). The coating was directly heated to 150 °C to enhance dipole mobility and alignment. The poling temperature was selected based on prior literature [24] and preliminary experimental trials. Although the Curie temperature of BIT is reported to be in the range of 650–770 °C, poling is typically conducted well below the Curie temperature to reduce the risk of dielectric breakdown under high electric field conditions. A DC electric field of 30 kV/cm was applied for 7 min. The applied voltage was determined using

(1) V=E·THK,

where *E* is the coercive field and THK is the measured coating thickness. Thickness was measured prior to poling, as described in Section 2.2.1. For field-deployed applications, thickness measurement could be performed using a micrometer. Literature reports coercive field values for BIT of approximately 36 kV/cm [25]. When electric fields approaching this value were applied in preliminary trials, dielectric breakdown occurred in several samples. Consequently, lower field levels were investigated to identify a stable operating range. Through parametric testing, an electric field of 30 kV/cm was found to provide reliable polarization without electrical failure. Therefore, 30 kV/cm was adopted as a conservative and empirically optimized poling field for all samples.

The poling duration of 7 min was established experimentally through parametric testing (5–10 min). It was observed that the measured $d_{33}$ increased up to approximately 7 min and then saturated, with no measurable improvement at longer durations. After poling, the samples were cooled to room temperature with the DC field turned off, resulting in remnant polarization.

### 2.2. Characterization of Transducers

#### 2.2.1. Thickness and Surface Roughness

The coating thickness is measured using a profilometer, i.e., the VK-X3100 confocal laser microscope (Keyence Corporation, Osaka, Japan) by comparing the height difference between a reference surface (without the coating) and the coated region. Accurate thickness measurement is essential to determine the appropriate poling voltage. Thickness also influences the transducer’s resonance frequency, making it a critical design parameter. In this study, the thickness of the BIT coating typically ranged from 35 to 80 µm. All measurements are made using the profilometer’s focus variation scan mode with a 5× objective lens.

**Figure 1:**
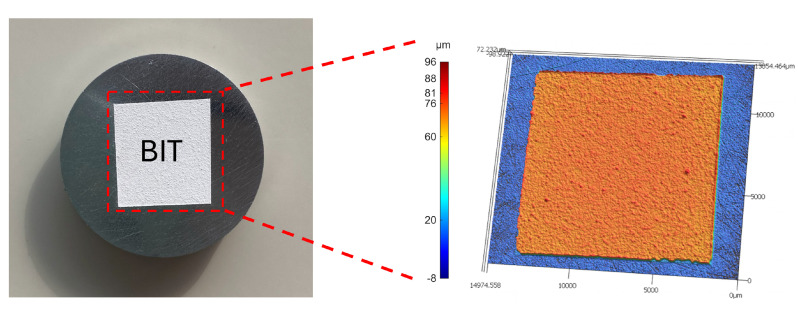
BIT coating sprayed onto 12.7 mm diameter by 6.35 mm thick stainless steel disk showing the surface profile of the BIT coating obtained by focus variation microscopy. Images obtained before applying the silver electrode.

For the transducers to have a high signal-to-noise ratio (SNR), the BIT thickness should be uniform. Nonuniform thickness would result in variable electric fields during poling and operation, as well as a range of resonance frequencies. The representative surface profile in Figure 1 is deemed sufficiently uniform, with an average thickness of approximately 81 μm, a maximum of 88 μm, and a minimum of 76 μm.

Surface roughness was measured with a KEYENCE optical profilometer in laser confocal mode using a 10× objective. Across multiple sprayed coatings and locations, the areal average roughness ($S_{a}$) ranged from 3.0 to 8.9 µm (mean 5.1 µm, std. dev. 1.9 µm). The RMS height ($S_{q}$) ranged from 5.1 to 11.7 µm (mean 6.6 µm, std. dev. 2.2 µm). The maximum height ($S_{z}$) ranged from 66.6 to 187 µm (mean 131.8 µm, std. dev. 49.0 µm). The surface roughness appears to be primarily a function of the particle size of the powder and agglomeration of the particles.

#### 2.2.2. Piezoelectric Coefficient

The piezoelectric coefficient $d_{33}$ of the transducers was measured using a commercial d33 m (PM300 PiezoMeter System, PIEZOTEST Pte. Ltd., Singapore) operating in HI/d33 mode. The measurements were performed using a static force of approximately 10–11 N, a dynamic force of 0.25 N, and a measurement frequency of 110 Hz. While this instrument is designed to test free-standing piezoelectric materials, our BIT coatings are deposited on stainless steel substrates; therefore, the measured values represent the effective response of the constrained coating–substrate system rather than that of a free-standing ceramic. The measurements are used for relative comparison between samples in our population.

#### 2.2.3. Pulse-Echo Ultrasound

Pulse-echo ultrasound measurements are carried out to evaluate the acoustic performance of the BIT transducers. Two ultrasonic systems are employed: the RAM 5000 (Ritec Inc., Warwick, RI, USA) and the TOPAZ 64 (Eddyfi Technologies, Quebec City, QC, Canada).

Using the RAM 5000 system, the transducers are driven with a 5-cycle sinusoidal tone burst having a prescribed central frequency. The central frequency is then swept over a broader frequency range to identify the resonance frequency from a forced response (similar to a frequency response function). The received echoes are recorded using the internal oscilloscope, and the amplitude of the first backwall echo is plotted as a function of frequency. This approach enables the determination of the resonance frequency as the point of maximum response. The RITEC amplifier drive level is set to 20, yielding approximately $120\;V_{\mathrm{pp}}$ at the 50 Ω port. Signals are averaged 64× and processed with a Butterworth band-pass filter centered at the excitation frequency $f_{c}$ with a 2 MHz bandwidth. The pulse-echo configuration also allows identification of successive backwall echoes, from which, knowing the substrate thickness, the sound velocity in the stainless steel substrate (∼$5900\;m\;s^{-1}$) was confirmed.

Using the TOPAZ 64 system, the transducers are excited with a negative square-wave voltage pulse duration of 60 ns and amplitude $100\;V_{\mathrm{pp}}$. The time-domain response (A-scan) is recorded, windowed, and Fourier transformed to obtain the frequency spectrum, which provides an independent estimate of the resonance frequency via the spectral peak of the transducer’s response. TOPAZ 64 acquisitions averaged signals together 16×, and a 3MHz high-pass filter was applied prior to spectral analysis.

## 3. Results

In this section results from pulse-echo testing of BIT transducers on stainless steel disks are presented for the pristine condition, after thermal exposure and after thermal cycling.

### 3.1. Ultrasonic Performance of Transducers As-Deposited

**Figure 2:**
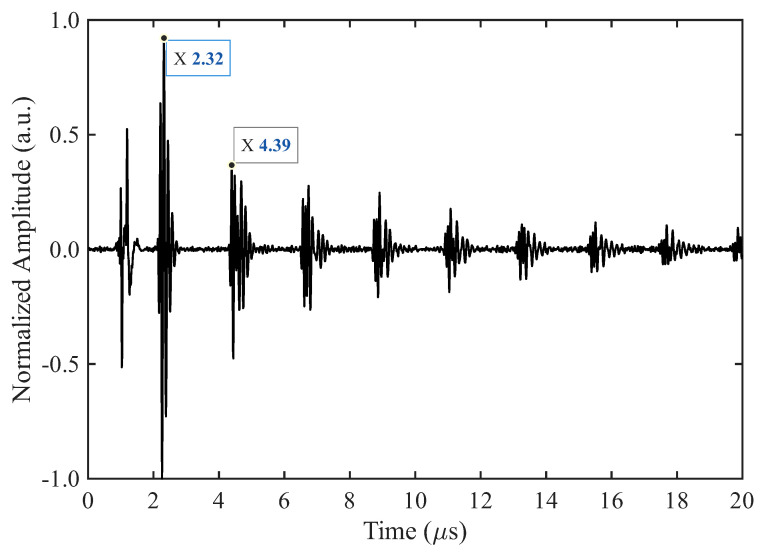
Representative pulse-echo signal received by TOPAZ 64, Sample BIT-11. Amplitude values were normalized by the maximum absolute peak amplitude ($2.89\times 10^{4}$ a.u.).

Pulse–echo tests enable us to characterize the resonance frequency, quality factor, and SNR of the transducers. Figure 2 shows a representative pulse-echo signal. The first peak in the received signal corresponds to electromagnetic interference rather than an ultrasonic wave. Thus, the second peak is the first backwall echo. The first backwall echo occurs at 2.32 μs, in close agreement with the expected value of 2.15 μs based on the longitudinal wave speed in stainless steel. The spacing between successive echoes is approximately 2.07 μs, which corresponds to the round trip time of flight of a longitudinal wave through the disk.

**Figure 3:**
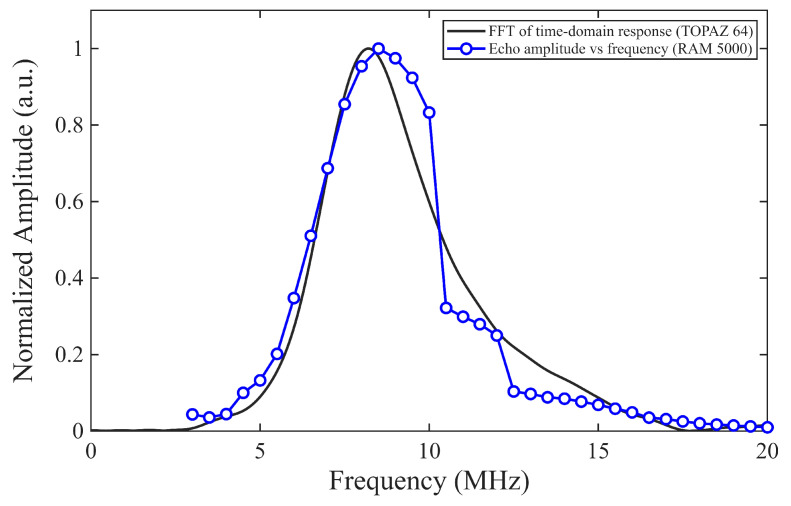
BIT transducer resonance frequency characterization. Transducer driven by square wave (TOPAZ 64) in one case and tonebursts having different center frequencies (RAM 5000) in the other, Sample BIT-11.

Resonance testing with an impedance analyzer [26] would not provide comparable results to a free-standing transducer due to the restraint of the disk. Instead, the resonance frequency isidentified by comparing the two characterization approaches described in Section 2.2.3. The frequency spectra obtained from the fast Fourier transform (FFT) of the TOPAZ 64 recorded and windowed time series and the frequency-dependent first backwall echo amplitude from the RAM 5000 system both show a clear resonance near 8.5 MHz in Figure 3.

**Figure 4:**
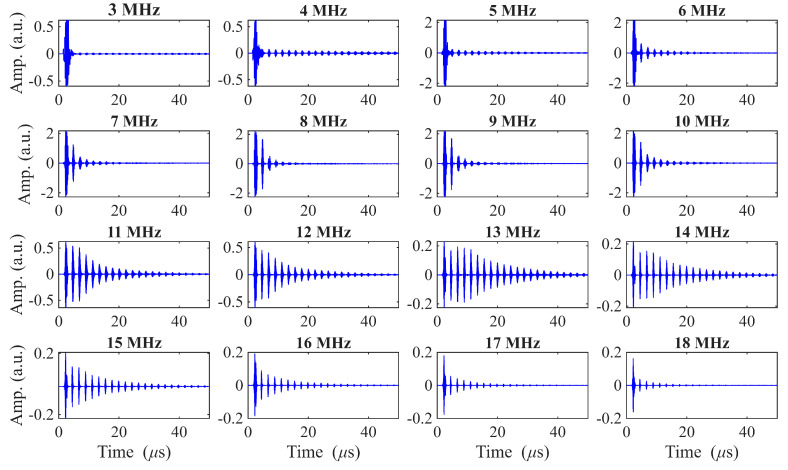
A-scans of pulse-echo ring-down signals for driving frequencies from 3–18 MHz (RAM 5000), Sample BIT-11. Each subplot is individually scaled in amplitude to enhance visualization of the signal features.

A-scans obtained with the RAM 5000 for center driving frequencies ranging from 3–18 MHz are shown in Figure 4. Notice that the amplitude axis range varies with frequency. Both the first backwall echo amplitude and the ring-down behavior are quite frequency-dependent, and the transducer appears to be quite broadband.

The frequency bandwidth of the transducers is characterized using the full width at half maximum (FWHM) of their resonance curves and the associated quality factor, $Q_{3\mathrm{dB}}$ [27]. The FWHM, also referred to as the −3 dB bandwidth, represents the frequency interval between the two points where the received power drops to half of its maximum value. The quality factor is defined as

(2) $$Q_{3\mathrm{dB}}=\frac{f_{0}}{\Delta f_{3\mathrm{dB}}},$$

where $f_{0}$ is the resonance frequency and $\Delta f_{3\mathrm{dB}}$ is the −3 dB bandwidth. A smaller bandwidth corresponds to a higher quality factor, indicating narrower resonance, whereas a broader bandwidth corresponds to a lower *Q*, which generally represents stronger damping and greater energy loss per oscillation cycle. In the underdamped harmonic oscillator model, the relationship between the quality factor and damping can be expressed as

(3) $$Q\approx \frac{\omega_{d}}{2\beta },$$

where $\omega_{d}$ is the damped natural frequency and β is the decay (absorption) coefficient [28,29,30]. Accordingly, a larger bandwidth (lower *Q*) indicates higher damping and a broader resonance.

The representative resonance curve in Figure 3 shows a peak at approximately $f_{0}=8.5$ MHz with a −3 dB bandwidth of $\Delta f_{3\mathrm{dB}}\approx 3$ MHz, resulting in a quality factor of $Q_{3\mathrm{dB}}\approx 2.8$. The relatively large bandwidth suggests that the transducers are highly damped and broadband, which can be advantageous for ultrasonic sensing applications.

**Figure 5:**
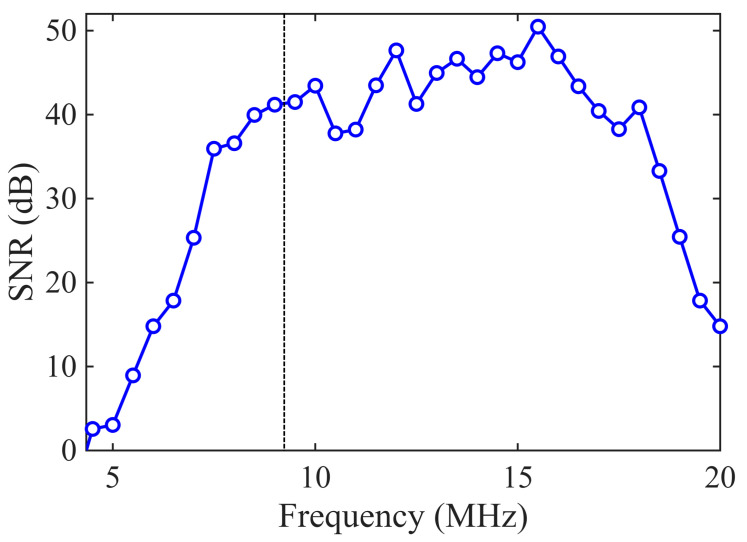
SNR of the first backwall echo as a function of driving frequency (RAM 5000), Sample BIT-11.

SNR is another important parameter that reflects the clarity of the signal received by the transducer. The BIT transducers exhibit SNR values in the range of 21–33 dB at resonance, which is higher than those reported in previous studies [21,24]. The SNR iscalculated using

(4) $$\mathrm{SNR}=20\log\left(\frac{V_{\mathrm{peak}}}{V_{\mathrm{noise}}}\right)$$

where $V_{\mathrm{peak}}$ is the peak amplitude of the first backwall echo and $V_{\mathrm{noise}}$ is the RMS amplitude measured in a noise-only window placed after the first backwall echo. Notably, in our results the SNR improved for frequencies above resonance. Although the signal amplitude generally decreases as the driving frequency diverges from resonance, the noise amplitude drops more rapidly, yielding a net increase in the SNR, as shown in Figure 4 and Figure 5.

To relate coating thickness to acoustic performance, both the operational center frequency and echo amplitude were analyzed as functions of BIT layer thickness. In general, thinner BIT coatings exhibited higher center frequencies and stronger echo amplitudes, indicating more efficient acoustic transmission and sensitivity (see Appendix A for details).

Table 2 summarizes the measured characteristics of the transducers processed under nominally identical conditions. The full set of results is provided in the Appendix A Table A1. Overall, these measurements demonstrate that the air-sprayed BIT transducers are functionally reproducible. All twenty BIT transducers exhibited clear ultrasonic responses, confirming that the process consistently produces operational devices. As discussed above, the BIT coating thickness is achieved by manually spraying many layers. The standard deviation is less than 9 μm (17%). The thickness deviation leads to center frequency deviation of 20%, while the amplitude deviation is larger, at 49%. Importantly, the transducers collectively show broadband capabilities as shown in Figure 4.

The measured $d_{33}$ values for the pristine transducer population ranged from 6 to 8 pC/N (Table 2 and Appendix A Table A1). Our measured values differ from those in the literature for two reasons: (i) the BIT coatings are not free standing and (ii) the BIT coatings are porous rather than dense sintered ceramics. For context, the reported $d_{33}$ value is 26 pC/N for bulk ceramic BIT [31]. Previous studies have shown that clamping effects can substantially reduce the measured $d_{33}$ of piezoelectric materials on substrates. For example, in clamped commercial PZT strips such as PZT-5H, the measured $d_{33}$ was reduced by approximately 62% due to the influence of the $d_{31}$ component during deformation [32]. This reduction arises from both direct and indirect piezoelectric effects that alter the effective displacement response.

### 3.2. Effect of Thermal Aging

Having established the characteristics of the transducer population (N = 20) in the as-processed state, a subset (N = 5) is thermally aged, a subset (N = 6) is thermally cycled, and an additional set of samples (N = 3) is pre-conditioned. The use of these small subsets is deemed to be warranted given the reproducibility of the transducer characteristics as-processed and the constraints on time and equipment. The performance of five representative transducers is evaluated following thermal aging in an argon-purged tube furnace at 375 °C for seven days. The selected transducers include two that gave high amplitudes (BIT-8 and BIT-12), one with intermediate performance (BIT-9), and two with low amplitudes (BIT-2 and BIT-13). This range enables us to assess how transducers across the performance spectrum respond to thermal exposure. The transducer characteristics before and after thermal aging are given in Appendix C Table A2, where the samples are listed in order of decreasing BIT thickness (THK) in the pristine condition. The most evident characteristic is the increase in center frequency after aging.

**Figure 6:**
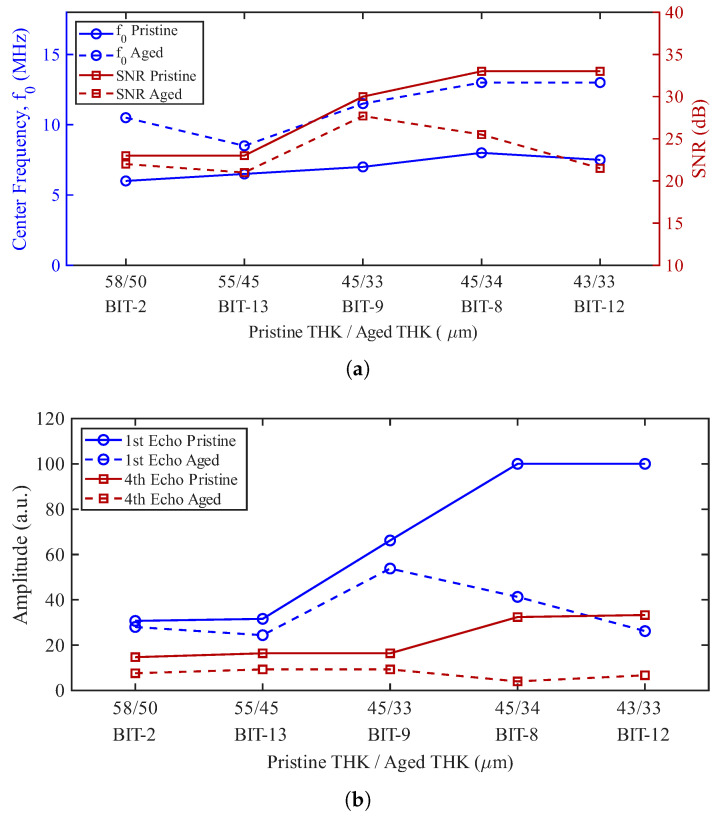
Comparison of acoustic metrics for BIT transducers before and after thermal aging: (**a**) center frequency, $f_{0}$ (left axis), and SNR (right axis) (**b**) amplitudes of the 1st and 4th backwall echoes.

The data in Appendix C Table A2 are plotted in Figure 6, which shows the center frequency, SNR, and 1st and 4th backwall echoes for both pristine and aged conditions of each transducer (arranged by decreasing BIT thickness). Clearly, the center frequency increases as BIT thickness decreases between the different transducers and due to aging. Likewise, the echo amplitudes are decreased by aging, especially for the thinner BIT coatings.

**Figure 7:**
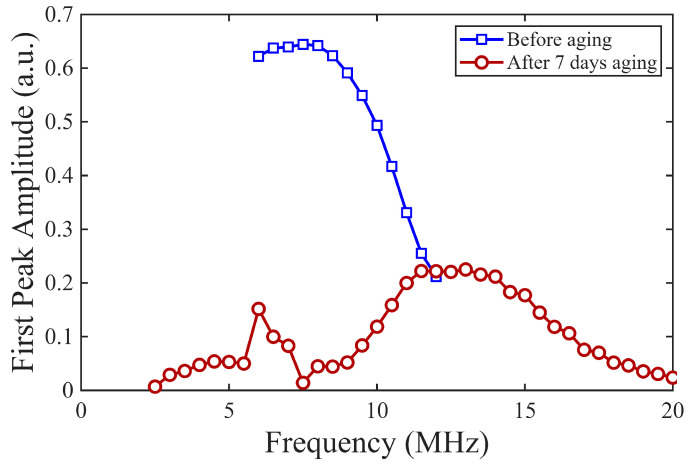
First backwall echo amplitude as a function of driving frequency (RAM 5000) before and after aging at 375 °C, Sample BIT-8.

Figure 7 shows the first backwall echo amplitude as a function of the (RAM 5000) driving frequency for one representative transducer (BIT-8). The peak of this curve, which is used here as a proxy for the resonance response (frequency of maximum received amplitude, see Section 2.2.3), shifts from approximately 8 to 13 MHz after aging. After thermal exposure, the BIT thickness was re-measured and found to decrease by approximately 8–12 μm across all samples. This reduction in thickness may explain the consistent increase in center frequency observed after aging.

To evaluate whether thickness reduction alone can explain the observed increase in resonance frequency, a first-order one-dimensional longitudinal resonance approximation was considered. Because the BIT coating is bonded to a stainless steel substrate, the system can be approximated as a clamped–free elastic layer for thickness-mode vibration. According to classical longitudinal vibration theory for a fixed–free bar [29], the natural frequencies scale as

$$f\propto \frac{1}{t}\sqrt{\frac{E}{\rho }},$$

where *t* is the coating thickness, *E* is Young’s modulus, and ρ is the mass density. Assuming *E* and ρ remain unchanged during thermal aging, the ratio of aged to pristine resonance frequency can be approximated as

$$\frac{f_{\mathrm{aged}}}{f_{\mathrm{pristine}}}\approx \frac{t_{\mathrm{pristine}}}{t_{\mathrm{aged}}}.$$

Using the measured thickness values in Table A2, thickness reduction alone predicts frequency increases on the order of 16–36%, depending on the sample. However, the experimentally observed increases range from approximately 31–75%. The larger measured shifts therefore suggest that thickness reduction is not the sole contributing factor and that changes in material properties may also influence the resonance response.

**Figure 8:**
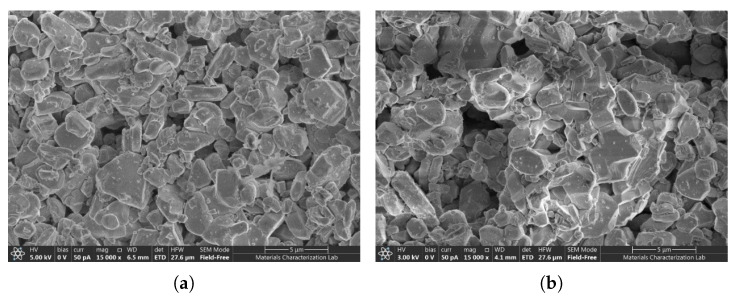
SEM images of the BIT coating: (**a**) pristine state and (**b**) aged state after exposure to 375 °C for seven days.

The BIT’s material properties (Young’s modulus and mass density) also affect the center frequency. We did not measure these properties, but we did examine the BIT for microstructural changes. Scanning electron microscopy (SEM) was performed using a Thermo Scientific^TM^ Axia^TM^ ChemiSEM (Thermo Fisher Scientific, Waltham, MA, USA) before and after thermal aging to investigate possible material changes. Representative images at 15,000× magnification of the BIT coating are shown in Figure 8. The BIT coating has a porous morphology both before and after aging. However, thermal exposure appears to cause particle agglomeration, resulting in the formation of some significantly larger pores. If the particle agglomeration increases the Young’s modulus, then it would increase the center frequency.

Additionally, to evaluate potential compositional changes due to high-temperature exposure, Energy-Dispersive X-ray Spectroscopy (EDX) is performed on the BIT coatings before and after thermal aging. EDX analysis is conducted using an SEM equipped with an Oxford Instruments X-Max detector at 15 kV acceleration voltage. Table 3 summarizes the elemental weight percentages obtained from pristine and aged samples. The results indicate that the elemental composition remained largely stable. Bismuth and titanium, key constituents of BIT, show only slight decreases in weight percentage following thermal exposure, while oxygen and carbon content increase moderately. These changes are likely attributable to partial surface oxidation and possible contamination during handling (e.g., the Si). The stability of the Bi, Ti, and O composition supports the chemical resilience of the BIT coating to this thermal exposure.

**Figure 9:**
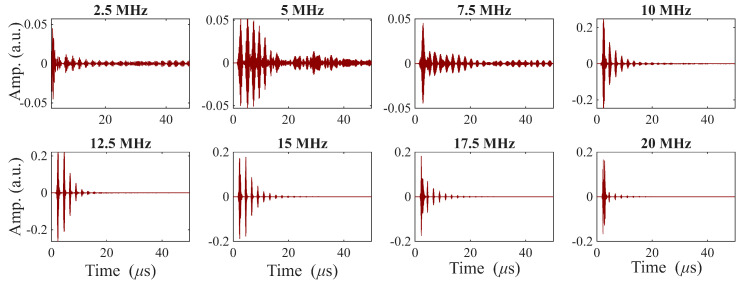
Pulse-echo measurements of BIT-8 transducer after thermal aging, driven at different frequencies (RAM 5000). Each subplot is individually scaled in amplitude to enhance visualization of the signal features.

Figure 9 shows pulse-echo signals for a range of driving frequencies that have at least four clearly distinguishable backwall echoes near its resonance frequency, which is true for all five transducers tested. The tabulated SNR results are shown in Figure 6 and Appendix C Table A2. Related to the SNR values are the amplitudes of the 1st and 4th echoes, which are shown in Figure 6b. Thermal aging reduces the echo amplitudes significantly for the thinner transducers (by a factor of roughly 3 for the 1st echo of BIT-12). A data-supported explanation for the loss of amplitude has eluded us. However, a plausible explanation is that the silver paste used for the electrode has been thermally damaged. On a more positive note, the range of amplitudes between the five transducers (which have a range of thicknesses) is reduced by a factor of two, meaning that there is less variance, essentially creating a beneficial normalizing effect.

Appendix C Table A2 shows that thermal aging increased $d_{33}$ from 6–8 pC/N in the pristine condition to 8–13 pC/N in the aged subset, consistent with previous studies on thermal treatment of ferroelectric ceramics [1,33]. This enhancement has been attributed to thermally induced microstructural changes such as domain reorientation, relaxation of internal stress fields, and grain growth [34].

**Figure 10:**
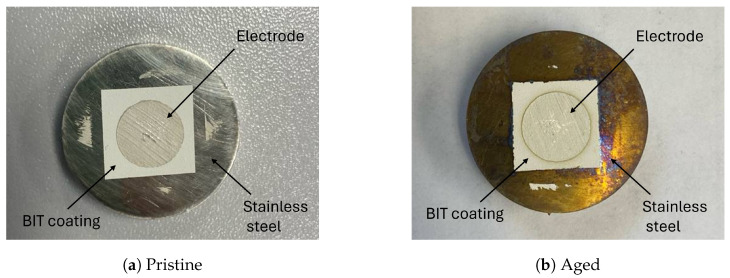

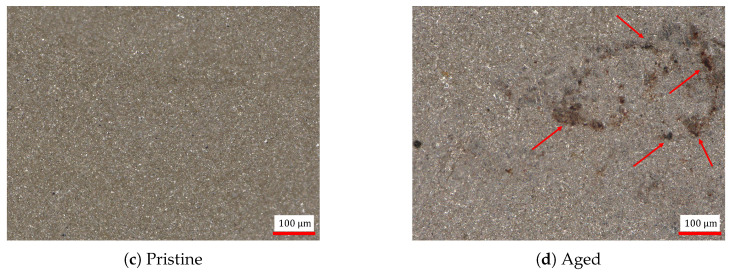
Macroscopic and microscopic appearance of BIT transducers before and after thermal aging. (**a**,**b**) Macroscopic views of the transducers bonded to the stainless steel disks with a diameter of 12.7 mm, showing no visible changes in surface texture between the pristine and aged conditions. (**c**,**d**) close-up images of the pristine and aged conditions.

While the observed increases in $d_{33}$ suggest enhanced piezoelectric performance, a noticeable reduction in the received signal amplitude is observed after thermal exposure. Despite this change in response, the macroscopic appearance of the transducers before and after thermal aging does not show significant differences or visible damage, as shown in Figure 10a,b. To further examine potential surface changes, optical microscopy was performed. In the microscopic images of the aged transducer (Figure 10d), subtle darkened regions or black fleck–like features, indicated by arrows, are observed on the electrode surface. These features may be indicative of thermally induced alterations in the electrode or its binder; however, they do not provide direct evidence of electrode degradation. Such changes could potentially contribute to the observed reduction in echo amplitude following thermal exposure.

### 3.3. Effect of Thermal Cycling

The effect of thermal cycling on the functionality of the transducers is evaluated. The transducers were placed on a hot plate; once the prescribed temperature of one sample’s surface was attained (a thermocouple was used), they were held in place for nominally 10 min and then removed to air-cool, before starting the next cycle. Three pristine samples and three thermally aged samples (exposed to 375 °C for seven days) were selected to undergo thermal cycling. Fifty cycles at 150 °C were followed by 50 cycles at 250 °C.

Pulse-echo tests were conducted during the initial 10 cycles to 150 °C, followed by additional measurements after every subsequent 10 cycles. After completing 50 thermal cycles to 150 °C, no significant variation in ultrasonic performance is observed for pristine or thermally aged transducers. This consistent behavior indicates good thermal stability at this moderate temperature. The amplitude and frequency content of the received signals remain nearly unchanged, indicating that the transducers are robust and stable under these conditions.

**Figure 11:**
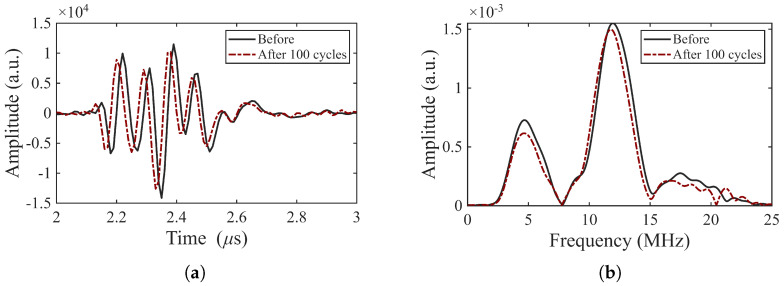
Pulse-echo response of an aged transducer (BIT-8): (**a**) A-scan of the first backwall echo and (**b**) corresponding frequency spectrum before and after 50 cycles to 150 °C plus 50 cycles to 250 °C (TOPAZ 64).

The signals from thermally aged samples remain stable after cycling to 250 °C, exhibiting only a slight decrease in amplitude, as shown in Figure 11. We conclude that aging at 375 °C for seven days enables these transducers to remain stable when cycled to a maximum temperature of 250 °C.

**Figure 12:**
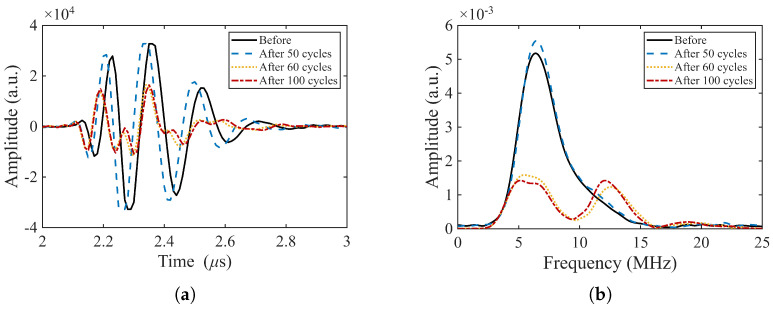
Evolution of the pulse-echo response of a pristine transducer (BIT–18) during thermal cycling (TOPAZ 64). (**a**) First backwall echo; (**b**) corresponding frequency spectra for the pristine condition and after successive thermal cycling stages: after 50 cycles to 150 °C followed by 10 cycles to 250 °C, and after 50 cycles to 150 °C followed by 50 cycles to 250 °C.

The signals from pristine samples remain stable over 50 cycles to 150 °C as shown in Figure 12. In contrast, subsequent cycling to 250 °C results in significant changes. After only 10 such cycles, the transducers exhibits a marked decrease in signal amplitude, accompanied by the appearance of an additional high frequency component in the spectrum. Up to the one hundredth cycle (50th at 250 °C), the overall signal amplitude and frequency remain relatively constant.

**Figure 13:**
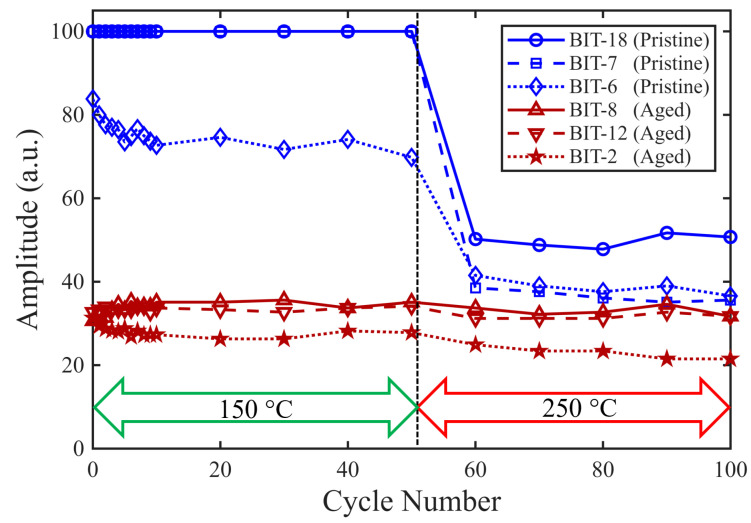
First backwall echo amplitude as a function of thermal cycles for all samples.

The results from all six samples subjected to thermal cycling are compiled in Figure 13, which plots the evolution of the amplitude of the first backwall echo over the applied thermal cycles. The only significant change in amplitude occurs for the three originally pristine samples after the peak temperature was increased to 250 °C, which implies that cycling is less important than the actual temperature. Thus, the primary driver of performance change in these tests is believed to be the temperature level, rather than the fluctuations. In summary, we find that thermal exposure above 150 °C changes the pristine transducer, and once aged at 375 °C, future exposures up to 250 °C (in these tests) do not significantly change the transducer.

### 3.4. Effect of Pre-Conditioning

The effects of thermal exposures described in the previous two sections suggest that thermal aging may pre-condition the transducers to have a more consistent and stable response. Thermal exposure alters the BIT morphology; particles tend to agglomerate, and the overall thickness decreases. In addition, it is possible that the Young’s modulus and mass density change. Therefore, we conducted a limited experiment to assess the effect of pre-conditioning. Three new BIT-coated samples were aged at 400 °C until their thicknesses and masses stabilized, reaching an apparent equilibrium condition after approximately 48 h. The results indicate a thickness reduction of 12–14% and a mass loss of roughly 11–17%. Details are provided in Table 4. The mass loss may reflect volatilization or some other form of binder removal. The mass loss, along with changes in porosity, may be responsible for thickness reduction. The apparent equilibrium condition is assumed to be associated with thermal stability.

**Figure 14:**
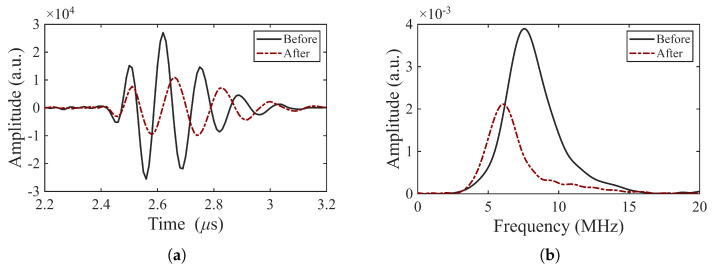
Pulse-echo response of the thermally pre-conditioned transducer BIT–23: (**a**) A-scan of the first backwall echo; (**b**) corresponding frequency spectrum.

Following pre-conditioning, electrodes were applied and the BIT coatings were poled and evaluated in pulse-echo mode as before. The transducers were subsequently thermally aged at 350 °C for 24 h and re-tested. The comparison of pulse-echo responses before and after thermal aging, shown in Figure 14, reveals a reduced echo amplitude after aging that may be associated with material changes in the silver electrode during thermal exposure (refer to Figure 10d). However, the resonance frequency does not increase after aging. Instead, a slight decrease in resonance frequency is observed. It should be noted that the measured BIT thickness after aging is the same as after pre-conditioning. Table 5 summarizes the change in resonance frequency of the pre-conditioned transducers before and after thermal aging. As the study of pre-conditioning is limited in scope, this will have to be addressed in future work.

## 4. Discussion

This work investigated the processing and characterization of air-sprayed bismuth titanate (Bi_4_Ti_3_O_12_, or BIT) ultrasonic transducers deposited on stainless steel disks. The transducer processing was done in a laboratory using field-deployable methodology. The raw materials and equipment are affordable and readily available. BIT powder was mixed with a ceramic binder and water to form a sprayable slurry. Multiple spray passes were made until the target thickness was achieved; e.g., 30 passes to yield a 60 μm thickness. The BIT coating has porosity. An electrode was added and the coating was poled to functionalize it. Twenty transducers were successfully processed and characterized. In these 20 transducers the BIT coating thicknesses ranged from 33 to 68 μm, providing center frequencies between 4.5 and 9.5 MHz in pulse-echo tests. The transducers are extraordinarily broadband and the signal-to-noise ratios range from 20–33 dB. Thus, we conclude that the processing methods used here are both successful and repeatable.

The Curie temperature of BIT is in the 650–770 °C range, making it suitable for elevated temperature applications. The subset of the BIT transducer population (5 of 20) thermally aged at 375 °C for seven days exhibited decreases in BIT thickness and changes in the microstructure (i.e., larger agglomerated particles and larger pores). Increases in the center frequency ranging from 30.8–75.0% (the mean is 61.2%) are associated with these changes. Likewise, the echo amplitudes decreased after thermal aging, although the signal-to-noise ratio did not suffer significantly. We suspect, but have not proven, that the decreased echo amplitudes are due to thermal damage to the binder in the silver paste electrode. Thus, choosing a more suitable electrode material could solve this problem. There are some applications where the measurements are immune to these changing response characteristics; thickness measurements, for example. However, other applications require thermally stable transducer characteristics.

Thermal cycling testing did not reveal steady degradation in transducer characteristics, but they did provide additional thermal tolerance information. First, the transducers were stable over 50 cycles between room temperature and 150 °C. Second, the transducers that were thermally aged at 375 °C were stable over another 50 cycles between room temperature and 250 °C. However, the pristine transducers were unstable over another 50 cycles between room temperature and 250 °C. In fact, changes occurred in the initial 250 °C cycles. Thus, 250 °C is sufficient to cause changes to BIT coatings.

Finally, it is evident from these results that thermal pre-conditioning could conceivably stabilize BIT transducers over a larger temperature range. A limited set of tests was conducted and, indeed, once pre-conditioned at 400 °C, there was no further decrease in BIT thickness when aged at 350 °C. There were decreased echo amplitudes, which is likely the same mechanism as discussed previously. Rather than an increase in the center frequency, there was actually a much smaller decrease in center frequency, which remains to be explained in future work. While there are still some questions to answer, air-sprayed BIT deposition paves the way for in-situ ultrasonic testing or continuous monitoring of materials in a variety of structural systems operating in harsh environments. Although the testing in the present work relied exclusively on pulse-echo mode, that was for convenience and is not a limitation.

## Figures and Tables

**Table 1:** Ultrasonic transducer comparisons. Abbreviations: COTS, commercial off-the-shelf; EMAT, electromagnetic acoustic transducer.

Type	Description	Advantages	Disadvantages
COTS contact transducers	Piezoelectric material potted in a protective housing; typically coupled with fluid or gel; piezoelectric element is usually PZT; typically used manually for inspection	A wide range of operating frequencies are available; proven designs; oblique incidence possible when mounted on a wedge	Limited operating temperature range; requires couplant and flat surfaces
Piezoelectric wafers	Thin piezoelectric layer bonded directly to the substrate for structural health monitoring; typically relies on in-plane resonances using $d_{31}$ coupling	Inexpensive; actuates and senses guided waves in plates	Limited operating temperature range; requires relatively flat surfaces; relies on bond integrity of the adhesive
Wave guide/delay lines	Use a wave guide to enable the transducer to operate in a less harsh environment	Enables PZT transducers to be used for higher temperature applications	There may be multiple modes in the wave guide; wave guide must be coupled at both ends
EMATs	Operate through the Lorentz force or magnetostriction; technically noncontact but lift-off is typically small	Versatile generation of wave types; harsh environment	Small lift-off; relatively small amplitude
Air-sprayed coatings (sol-gel process), e.g., [14]	Piezoelectric powder deposited by sol-gel process and then heated	Curved surfaces; no couplant; high Curie temperature	Deposition and high temperature sintering and poling in laboratory
Air-sprayed coatings (ceramic binder), e.g., this article	Piezoelectric powder mixed into ceramic binder	Curved surfaces; no couplant; high Curie temperature; field deposition	Limited data

**Table 2:** Statistical summary of 20 BIT transducers.

Parameter	Mean	Std. Dev.	Min	Max
THK (μm)	51.05	8.77	33	68
Center Frequency (MHz) *	7.13	1.46	4.5	9.5
SNR (dB)	28.13	4.54	20	33
1st Echo Amp. (a.u.)	62.12	30.40	21.8	100
4th Echo Amp. (a.u.)	23.22	11.21	10.2	55.6
A4/A1 (–)	0.41	0.15	0.16	0.63
$d_{33}$ (pC/N)	7.20	0.70	6	8

* Center frequency statistics are based on 15 representative transducers.

**Table 3:** EDX elemental composition (wt%) of BIT coatings before and after thermal aging at 375 °C for seven days.

Element	Pristine	Aged	Difference
Bi	66.89	63.23	−3.66
O	15.72	18.34	+2.62
Ti	11.40	10.33	−1.07
C	5.64	7.58	+1.94
Si	0.35	0.52	+0.17

**Table 4:** Thickness and mass loss of BIT coating after pre-conditioning at 400 °C for 48 h.

Sample	Thickness (μm)	Mass Loss (mg)	Mass Loss (%)
BIT-21	56 → 48	4.4	11.4
BIT-22	56 → 48	6.2	14.6
BIT-23	55 → 49	6.0	17.1

**Table 5:** Resonance frequency of pre-conditioned transducers before and after thermal aging at 350 °C.

Sample	Before Aging (MHz)	After Aging (MHz)	Δ*f* (%)
BIT-21	6.88	5.47	−20.5
BIT-22	7.18	5.96	−17.0
BIT-23	7.52	5.96	−20.7

**Figure A1:**
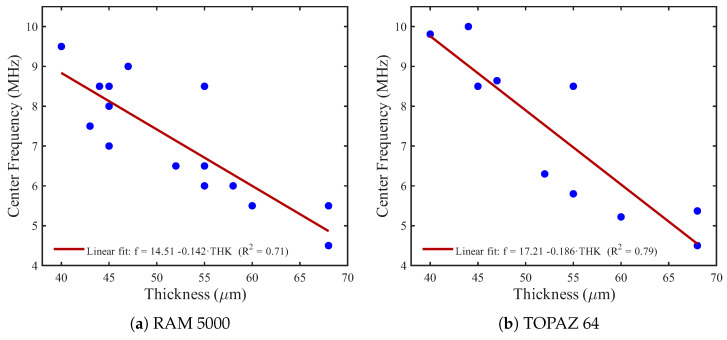
Center frequency versus BIT coating thickness for the two measurement methods.

**Figure A2:**
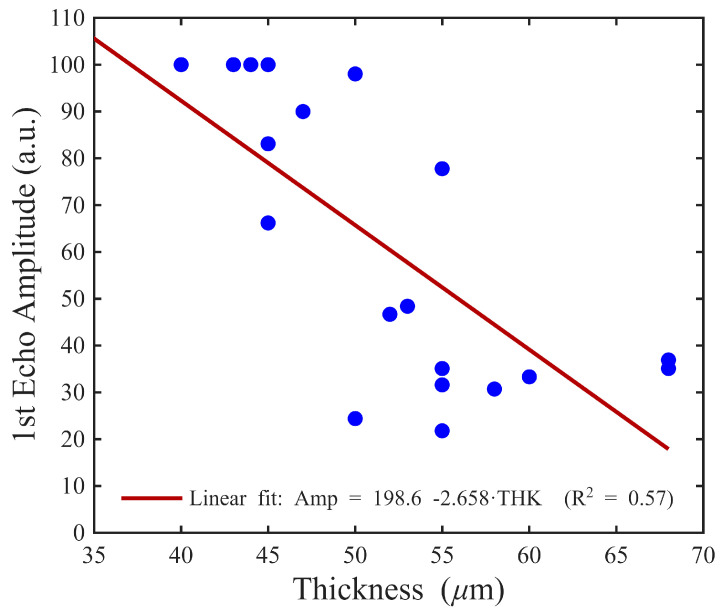
Dependence of pulse-echo sensitivity on BIT coating thickness.

## Data Availability

Data is contained within the article.

## Abbreviations

The following abbreviations are used in this manuscript:

BIT	Bismuth titanate (Bi_4_Ti_3_O_12_)
$d_{33}$	Piezoelectric coefficient
SNR	Signal-to-noise ratio
Q	Quality factor

## Appendix A. Relation Between Coating Thickness and Acoustic Performance

To relate coating thickness to acoustic performance, both the operational center frequency and the echo amplitude were analyzed as functions of BIT layer thickness. Figure A1 shows that the center frequency increases as the BIT thickness decreases for both the RAM 5000 and TOPAZ 64 instruments. For the RAM 5000 measurements, 15 representative transducers were included in the analysis, whereas for the TOPAZ 64 system, 10 transducers were analyzed due to measurement availability. This trend was also observed in the thermal aging results discussed in Section 3.2.

Figure A2 shows that the first-echo amplitude nominally increases as coating thickness decreases. In other words, thinner transducers tend to produce stronger echo amplitudes, which can be attributed to their lower effective mass and mechanical impedance, resulting in more efficient acoustic transmission and higher sensitivity [35].

## Appendix B. Summary of 20 BIT Transducers

Table A1 provides the full set of measured characteristics for the population of 20 BIT transducers processed under nominally identical conditions. The characteristics include coating thickness (THK), center frequency (results not available for five samples), SNR, first and fourth echo amplitudes, amplitude ratio (A4/A1), and piezoelectric coefficient ($d_{33}$). Ultrasound characteristics are based on TOPAZ 64 measurements.

**Table A1:** Characteristics of BIT transducer population (TOPAZ).

Sample	THK	Center Freq.	SNR	1st Echo	4th Echo	A4/A1	d_33_
	(μm)	(MHz)	(dB)	(a.u.)	(a.u.)	(–)	(pC/N)
BIT-1	68	5.50	24.0	35.1	24.0	0.60	7
BIT-2	58	6.00	23.0	30.7	14.7	0.47	8
BIT-3	53	–	27.0	48.4	30.7	0.63	8
BIT-4	50	–	21.0	24.4	15.6	0.63	7
BIT-5	55	–	20.0	21.8	12.0	0.55	8
BIT-6	55	6.00	24.0	35.1	19.1	0.54	7
BIT-7	45	8.50	31.5	83.1	25.3	0.30	7
BIT-8	45	8.00	33.0	100.0	32.4	0.32	8
BIT-9	45	7.00	30.0	66.2	16.4	0.24	7
BIT-10	50	–	33.0	98.0	37.8	0.38	7
BIT-11	44	8.25	33.0	100.0	55.6	0.55	8
BIT-12	43	7.50	33.0	100.0	33.3	0.33	6
BIT-13	55	6.50	23.0	31.6	16.4	0.51	6
BIT-14	55	8.50	31.0	77.8	34.2	0.44	7
BIT-15	33	–	33.0	99.0	20.0	0.20	8
BIT-16	60	5.50	27.0	33.3	15.6	0.47	7
BIT-17	47	9.00	31.0	74.2	12.0	0.16	6
BIT-18	52	6.50	27.0	46.7	20.9	0.45	7
BIT-19	68	4.50	25.0	36.9	10.2	0.27	8
BIT-20	40	9.50	33.0	100.0	18.2	0.18	7

## Appendix C. BIT Transducer Characteristics Before and After Thermal Aging

Table A2 provides the complete set of measurements for the five representative BIT transducers evaluated before and after thermal aging. These data include coating thickness, center frequency, signal-to-noise ratio (SNR), first- and fourth-echo amplitudes, amplitude ratio (A4/A1), and piezoelectric coefficient ($d_{33}$).

**Table A2:** BIT transducer characteristics before and after thermal aging (TOPAZ 64).

Sample	State	THK	Center Freq.	SNR	1st Echo	4th Echo	A4/A1	d_33_
		(μm)	(MHz)	(dB)	(a.u.)	(a.u.)	(–)	(pC/N)
BIT-2	Pristine	58	6.0	23.0	30.7	14.7	0.47	8
	Aged	50	10.5	22.0	28.0	7.6	0.27	9
BIT-13	Pristine	55	6.5	23.0	31.6	16.4	0.51	6
	Aged	45	8.5	21.0	24.4	9.3	0.38	10
BIT-9	Pristine	45	7.0	30.0	66.2	16.4	0.24	7
	Aged	33	11.5	27.7	53.8	9.3	0.17	13
BIT-8	Pristine	45	8.0	33.0	100.0	32.4	0.32	8
	Aged	34	13.0	25.5	41.3	4.0	0.09	11
BIT-12	Pristine	43	7.5	33.0	100.0	33.3	0.33	6
	Aged	33	13.0	21.5	26.2	6.7	0.25	8
